# Low-Molecular-Weight Collagen Peptide Ameliorates Osteoarthritis Progression through Promoting Extracellular Matrix Synthesis by Chondrocytes in a Rabbit Anterior Cruciate Ligament Transection Model

**DOI:** 10.4014/jmb.2108.08027

**Published:** 2021-09-11

**Authors:** Mun-Hoe Lee, Hyeong-Min Kim, Hee-Chul Chung, Do-Un Kim, Jin-Hee Lee

**Affiliations:** Health Food Research and Development, NEWTREE Co., Ltd., Seoul 05604, Republic of Korea

**Keywords:** Osteoarthritis, ACLT rabbit, chondrocyte, collagen hydrolysate, low-molecular-weight collagen peptide, cartilage regeneration

## Abstract

This study examined whether the oral administration of low-molecular-weight collagen peptide (LMCP) containing 3% Gly-Pro-Hyp with >15% tripeptide (Gly-X-Y) content could ameliorate osteoarthritis (OA) progression using a rabbit anterior cruciate ligament transection (ACLT) model of induced OA and chondrocytes isolated from a patient with OA. Oral LMCP administration (100 or 200 mg/kg/day) for 12 weeks ameliorated cartilage damage and reduced the loss of proteoglycan compared to the findings in the ACLT control group, resulting in dose-dependent (*p* < 0.05) improvements of the OARSI score in hematoxylin & eosin (H&E) and Safranin O staining. In microcomputed tomography analysis, LMCP also significantly (*p* < 0.05) suppressed the deterioration of the microstructure in tibial subchondral bone during OA progression. The elevation of IL-1β and IL-6 concentrations in synovial fluid following OA induction was dose-dependently (*p* < 0.05) reduced by LMCP treatment. Furthermore, immunohistochemistry illustrated that LMCP significantly (*p* < 0.05) upregulated type II collagen and downregulated matrix metalloproteinase-13 in cartilage tissue. Consistent with the in vivo results, LMCP significantly (*p* < 0.05) increased the mRNA expression of *COL2A1* and *ACAN* in chondrocytes isolated from a patient with OA regardless of the conditions for IL-1β induction. These findings suggest that LMCP has potential as a therapeutic treatment for OA that stimulates cartilage regeneration.

## Introduction

Osteoarthritis (OA) is a degenerative arthritis disease mainly caused by aging, and it is accompanied by joint pain and stiffness as symptoms. More than 250 million patients have OA globally, and the disease affects 12% of elderly people in Western countries [1]. Because pain and movement disorders in daily life caused by OA reduce patient quality of life and result in astronomical social costs for treatment, preventing and effectively treating OA are extremely meaningful from a public health perspective. The main clinical treatments for OA are analgesic or nonsteroidal anti-inflammatory drugs, but the long-term use of these drugs leads to side effects [2, 3]. To overcome these problems, food-grade safe ingredients are being developed [4]. Recently, studies have attempted to suppress OA via the oral administration of cartilage components such as glucosamine hydrochloride, mucopolysaccharide–protein complexes, and hyaluronic acid [5, 6].

Collagen is a primary extracellular matrix (ECM) substance in cartilage, and it is produced by chondrocytes [7]. Collagen has long been used as a medical material because it promotes tissue regeneration by stimulating major cells in collagen-rich tissues [8]. In recent years, it was reported that the oral administration of collagen hydrolysate (CH) obtained by hydrolyzing collagen through enzyme engineering has various health benefits in humans including the prevention of skin aging and stimulation of bone formation and cartilage regeneration through ECM synthesis in cells [9]. Tsuruoka *et al*. [10] confirmed that CH stimulates bone fracture healing by promoting type I collagen synthesis in osteoblastic cells. Additionally, periodic knee injections of CH stimulated type II collagen synthesis and suppressed matrix metalloproteinase 13 (MMP13) expression in chondrocytes, thereby alleviating OA in a rabbit anterior cruciate ligament transection (ACLT) model [11]. Among the various peptide sequences included in CH, Gly-Pro-Hyp and Pro-Hyp are the major functional components. It was reported that Gly-Pro-Hyp accelerates osteoblastic proliferation and differentiation in MC3T3-E1 cells, and Pro-Hyp stimulates skin fibroblast growth [12, 13]. Both bioactive peptides are absorbed into blood plasma in a partially intact form and delivered to tissues following oral administration [14]. In the process of CH absorption, even if Pro-Hyp is not present in the consumed CH, other peptides are decomposed and absorbed as Pro-Hyp. However, Gly-Pro-Hyp is rarely obtained from the decomposition of other peptides during absorption [15]. Therefore, to effectively absorb both peptides, it is recommended to consume CH containing Gly-Pro-Hyp.

Low-molecular-weight collagen peptide (LMCP) is a form of CH derived from fish skin containing 3% Gly-Pro-Hyp, with a tripeptide content exceeding 15%. LMCP is individually recognized as a health functional food ingredient for skin function by the Ministry of Food and Drug Safety in the Republic of Korea. In our previous study, oral LMCP administration displayed health benefits in human skin by reducing wrinkles and increasing hydration and elasticity [16]. Through a mechanism study, we confirmed that LMCP stimulated skin fibroblasts to synthesize type I collagen and hyaluronic acid [17, 18]. In addition, LMCP inhibits the decomposition of dermal collagen by downregulating the gene expression of collagenases (MMP-3 and MMP-13) and gelatinases (MMP-2 and MMP-9) [19]. Therefore, in this study, we hypothesized that LMCP could alleviate OA by stimulating chondrocytes in cartilage to synthesize collagen fibers and suppress MMP13 expression as it acts on the skin. To confirm the effect of LMCP on OA progression, we used a rabbit ACLT model of induced OA and primary chondrocytes isolated from a patient with OA.

## Materials and Methods

### Test Material

LMCP (NEWTREE Co., Ltd., Korea), which is a form of CH obtained from the skin of *Pangasius hypophthalmus* containing 3% Gly-Pro-Hyp and a tripeptide content exceeding 15%, was used in our in vitro and in vivo experiments. LMCP was dissolved in distilled water before use.

### Rabbit ACLT Model of Induced OA

Thirty-two New Zealand white rabbits (2.5–3.0 kg; male) were purchased from Hallym Laboratory Animal Research Center (Hwaseong-si, Gyeonggi-do, South Korea) and maintained in an animal facility with controlled conditions (temperature, 23°C ± 3°C; relative humidity, 55% ± 15%; ventilation frequency, 10–20 times/h; lighting time, 12 h [8 am–8 pm]; and illuminance, 150–300 Lux [IACUC Approval No. 20-KE-404]). After 1 week of acclimation, ACLT surgery was performed on the right joint of rabbits to induce OA. Briefly, after anesthetizing the animals, the right knee periphery was epilated using clippers. The surgical site was widely disinfected with povidone and 70% alcohol, and the skin was incised. The surrounding tissue was subjected to blunt dissection to expose the articular surface at the distal end of the right femur. After cutting the anterior cruciate ligament with surgical scissors, the wound was sutured using 4-0 nylon. After the induction of OA, antibiotics (cephradine), and analgesics (tramadol) were administered for 3 days to suppress inflammation caused by surgery [20].

One week after surgery, rabbits were separated into four groups (*n* = 8), and LMCP or distilled water was orally administered for 12 weeks. The sham and ACLT groups were administered distilled water, and the 100 LMCP group and 200 LMCP group were administered 100 and 200 mg/kg bw/day LMCP. Rabbits were given free access to food and water during the experiments. At the end of the experiment, rabbits were fasted for 12 h before synovial fluid collection and euthanasia. The tissues of the ACLT surgical site were fixed in 10% neutral buffered formalin solution.

### Micro-Computed Tomography (CT) Analysis

The microstructure of femoral and tibial subchondral bone was measured using a micro-CT system (Scanco Medical, Bassersdorf, Switzerland) with the following parameters: voltex size, 60 μm; energy source, 55 kVp; intensity, 145 μA; and integration time, 150 ms. From the growth plate to the tip of each bone, an area corresponding to 154 slices for the femur and 84 slices for the tibia was set for measurement. For quantitative analysis, trabecular bone volume per total volume (BV/TV), trabecular bone surface per bone volume (BS/BV), trabecular bone number (TbN), trabecular separation, and trabecular bone thickness (TbTh) were analyzed.

### Biochemical and Histological Analyses

The concentrations of IL-1β and IL-6 in synovial fluid were analyzed using commercial ELISA kits (IL-1β, Cusabio, CSB-E06900Rb, Country; IL-6, RayBiotech, ELL-IL-6-1, USA) according to the instruction manual.

Fixed tissues were decalcified in commercial unbuffered 10% EDTA decalcification solution (Milestone, 51413G, USA) and embedded in paraffin. Paraffin-embedded tissue was sectioned to a thickness of 4 μm and stained with H&E or Safranin O according to standard protocols. The degree of OA progression was evaluated using the methods of Osteoarthritis Research Society International (OARSI) as described previously [21, 22]. To perform immunohistochemistry (IHC), the sectioned specimens were stained with antibodies against type II collagen (Sigma-Aldrich, CP18-100UGCN, USA) and MMP13 (Invitrogen, MA5-14238, USA) according to standard protocols.

### Isolation of Chondrocytes from the Cartilage of a Patient with OA

To isolate chondrocytes, we obtained cartilage samples from a patient with OA during knee cartilage replacement [23]. The cartilage tissue was sliced in serum-free (SF)-Dulbecco’s modified Eagle’s medium (DMEM) containing 0.5 mg/ml hyaluronidase (Sigma-Aldrich, H3506). The sliced tissue was incubated in SF-DMEM containing 5 mg/ml protease (Sigma-Aldrich, P5147) at 37°C for 1 h. The tissue was washed three times with SF-DMEM and then incubated in SF-DMEM containing 2 mg/ml collagenase (Sigma-Aldrich, C9263, USA) at 37°C for 3 h. Finally, the tissue was filtered through a 40-μm cell strainer (Corning, 352340, USA) to obtain a chondrocyte suspension. Cell suspensions were cultured at 1.3 × 10^4^ cells/cm^2^ in DMEM containing 10%inactivated fetal bovine serum (Gibco, 16000044, USA). Subculture was performed once a week and cells were used only when the passage number was less than 4.

### Cytotoxicity and Quantitative Real-Time Polymerase Chain Reaction (qRT-PCR)

Chondrocytes isolated from a patient with OA were seeded in 96- or 24-well plates at 1.3 × 10^4^ cells/cm^2^ and cultured for 24 h. For starvation, the medium was replaced with SF-DMEM, and cells were cultured for 16 h. Then, the chondrocytes were treated with various concentrations of LMCP (25–1,000 μg/ml) with or without 20 ng/ml recombinant human IL-1β (R&D Systems, 201-LB-025, USA) for 24 h. The group treated with distilled water and without 20 ng/ml recombinant human IL-1β was the CON group, and the group treated with distilled water and 20 ng/ml recombinant human IL-1β was the IL-1β group. To evaluate cytotoxicity, a CCK-8 kit (Dojindo, CK04-11, Korea) was used according to the instruction manual. To investigate mRNA expression, total RNA from chondrocytes was extracted using TRIzol reagent (Molecular Research Center, TR 118, USA) and reverse-transcribed into cDNA using a cDNA synthesis kit (Dyne Bio, BN615, Korea). qRT-PCR was performed using SYBR Green (Bioline, BIO-92020, UK). The sequences of primers used in this experiment are provided in Table 1.

### Statistical Analyses

All statistical analyses were performed using SPSS v26.0 software (SPSS, Inc., USA), and the results were expressed as the mean ± SD. Differences were analyzed using a *t*-test or one-way analysis of variance, followed by Duncan’s test.

## Results

### LMCP Ameliorated Cartilage Damage Associated with OA in the Rabbit ACLT Model

**Fig. 1:**
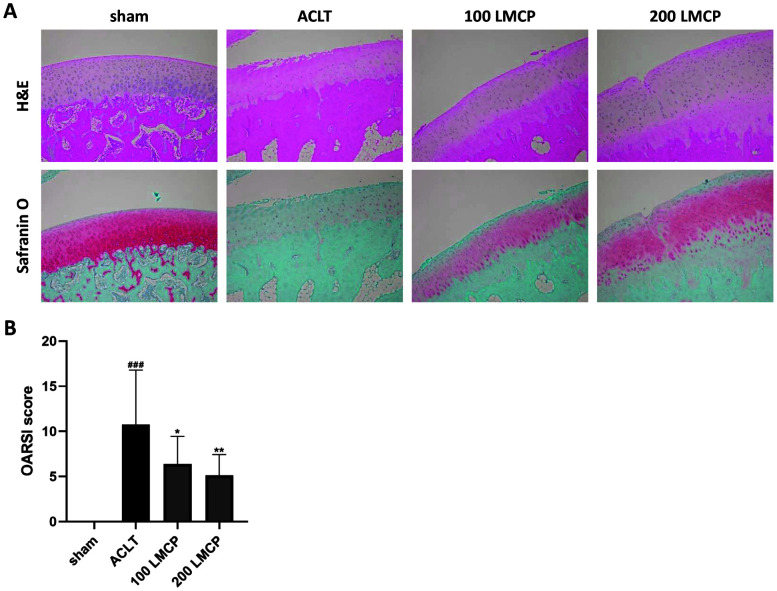
Low-molecular-weight collagen peptide (LMCP) ameliorated cartilage damage associated with osteoarthritis in the anterior cruciate ligament transection (ACLT) model. Rabbits underwent ACLT or sham operation and received treatment with vehicle or LMCP at 100 (100 LMCP) or 200 mg/kg (200 LMCP) by oral gavage once daily for 12 weeks. (**A**) Representative hematoxylin and eosin (H&E) and Safranin O images of cartilage (magnification, ×100). (**B**) OARSI score of cartilage. Data are presented as the mean ± SD (*n* = 8), and data were analyzed via one-way analysis of variance followed by Duncan’s test. ^###^*p* < 0.001 compared with the sham group. *p < 0.05 and **p < 0.01 compared with the ACLT group.

To investigate the protective effect of LMCP on cartilage, LMCP was orally administered to rabbits with ACLT-induced OA for 12 weeks. Cartilage damage was evaluated through H&E and Safranin O staining, which accurately confirms cartilage degeneration during OA progression [24]. According to the H&E staining (Fig. 1A), the cartilage surface was damaged and hypocellularity occurred following OA induction. In addition, Safranin O staining revealed severe proteoglycan loss in the ACLT group compared to the findings in the sham group. However, rabbits in the 100 LMCP and 200 LMCP groups had a smoother cartilage surface than those in the ACLT group. Hypocellularity in ACLT rabbits caused by OA was also alleviated by LMCP. Furthermore, the loss of proteoglycan was much less severe in the LMCP groups than in the ACLT group. Meanwhile, the OARSI score (Fig. 1B), which was increased following OA induction, was dose-dependently decreased by LMCP (*p* < 0.05). These results suggested that LMCP exerts protective effects on cartilage in this OA model.

### LMCP Inhibited OA-Associated Subchondral Bone Damage in the Rabbit ACLT Model

**Fig. 2:**
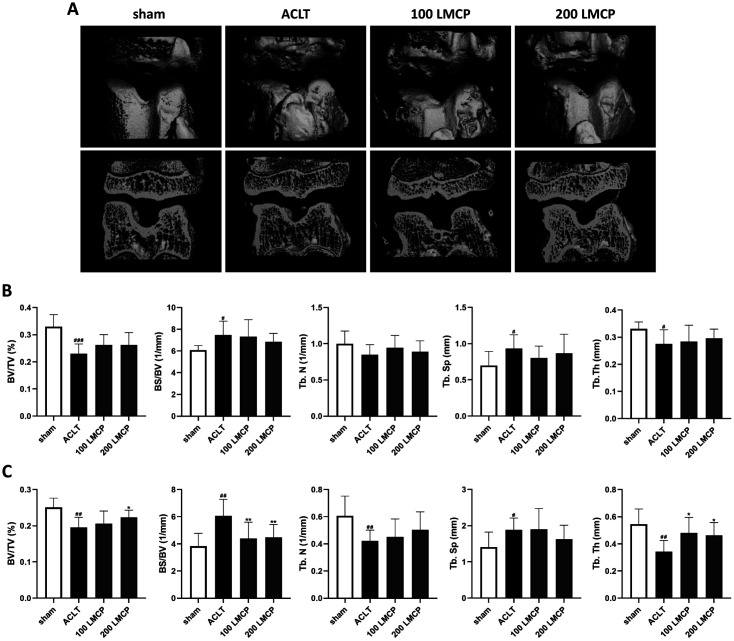
Low-molecular-weight collagen peptide (LMCP) inhibited subchondral bone damage associated with osteoarthritis in the rabbit anterior cruciate ligament transection (ACLT) model. Rabbits underwent ACLT or sham operation and received treatment with vehicle or LMCP at 100 (100 LMCP) or 200 mg/kg (200 LMCP) by oral gavage once daily for 12 weeks. (**A**) Representative micro-computed tomography (CT) images of subchondral bone. Quantitative micro-CT analysis of femoral (**B**) and tibial (**C**) subchondral bone. BV/TV, trabecular bone volume per total volume; BS/BV, trabecular bone surface per bone volume; TbN, trabecular number; TbSp, trabecular separation; TbTh, trabecular thickness. Data are presented as the mean ± SD (*n* = 8), and were analyzed via one-way analysis of variance followed by Duncan’s test. ^#^*p* < 0.05, ^##^*p* < 0.01, and ^###^*p* < 0.001 compared with the sham group. *p < 0.05 and **p < 0.01 compared with the ACLT group.

To examine subchondral bone damage that occurs after cartilage damage during OA, changes in the morphological microstructure of subchondral bone were analyzed using micro-CT. Biomarkers of the morphological microstructure of bone in the ACLT group were deteriorated compared to the findings in the sham group in both femoral and tibial subchondral bone (Figs. 2B, 2C). Meanwhile, LMCP significantly (*p* < 0.05) improved BV/TV, BS/BV, as well as the TbTh of tibial subchondral bone. In addition, the number of osteophytes in the ACLT group, which was severely increased by bone damage, was reduced in the 200 LMCP group (Fig. 2A). These data indicate that LMCP could inhibit bone damage caused by cartilage damage in OA.

### Effect of LMCP on Pro-Inflammatory Cytokines in Synovial Fluid in the Rabbit ACLT Model

**Fig. 3:**
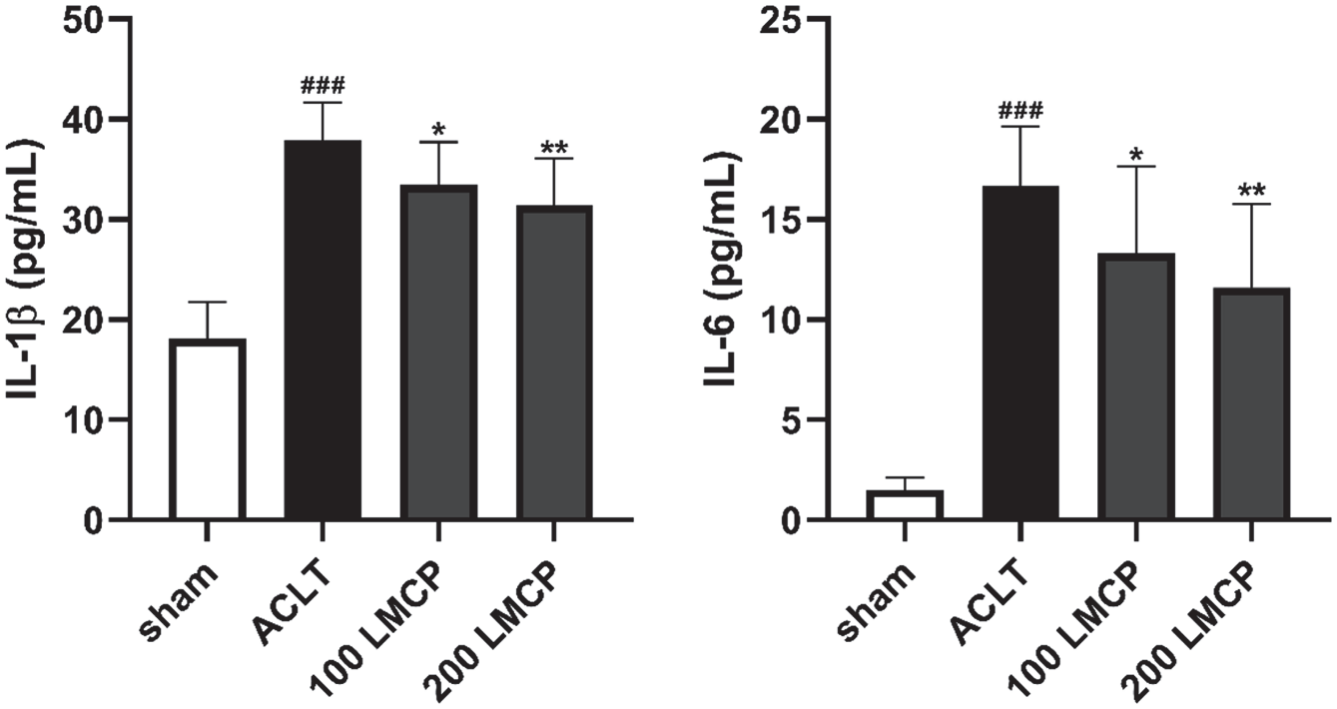
Inhibitory effect of low-molecular-weight collagen peptide (LMCP) on pro-inflammatory cytokines in synovial fluid in the rabbit anterior cruciate ligament transection (ACLT) model. Rabbits underwent ACLT or sham operation and received treatment with vehicle or LMCP at 100 (100 LMCP) or 200 mg/kg (200 LMCP) by oral gavage once daily for 12 weeks. Data are presented as the mean ± SD (*n* = 8), and were analyzed via one-way analysis of variance followed by Duncan’s test. ^###^*p* < 0.001 compared with the sham group. *p < 0.05 and **p < 0.01 compared with the ACLT group.

To determine the effect of LMCP on pro-inflammatory cytokines, the concentrations of IL-1β and IL-6, the major pro-inflammatory cytokines in OA, in synovial fluid were measured using commercial ELISA kits. As presented in Fig. 3, LMCP dose-dependently (*p* < 0.05) decreased the concentrations of IL-1β and IL-6, which were increased by OA induction. These data indicate that LMCP could effectively suppress the production of pro-inflammatory cytokines during OA development.

### Effect of LMCP on the Expression of Type II Collagen and MMP13 in Cartilage in the Rabbit ACLT Model

**Fig. 4:**
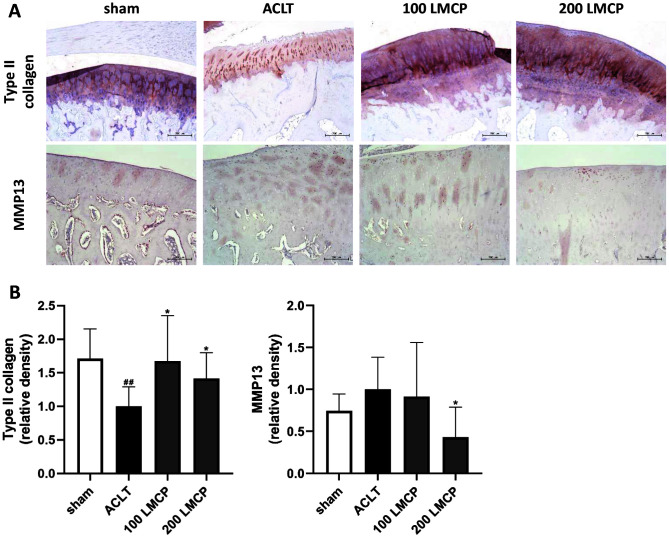
Effect of low-molecular-weight collagen peptide (LMCP) on the expression of type II collagen and matrix metalloproteinase 13 (MMP13) in cartilage in the rabbit anterior cruciate ligament transection (ACLT) model. Rabbits underwent ACLT or sham operation and received treatment with vehicle or LMCP at 100 (100 LMCP) or 200 mg/kg (200 LMCP) by oral gavage once daily for 12 weeks. (**A**) Representative immunohistochemical images of cartilage (magnification, ×100). (**B**) Relative density of type II collagen and MMP13 staining. Data are presented as the mean ± SD (*n* = 8), and were analyzed via one-way analysis of variance followed by Duncan’s test. ^##^*p* < 0.01 compared with the sham group. *p < 0.05 compared with the ACLT group.

Our data revealed the protective effect of LMCP on OA development. Therefore, to elucidate the molecular biological mechanism of LMCP, type II collagen and MMP13 expression in cartilage was analyzed using IHC (Fig. 4). LMCP significantly (*p* < 0.05) increased the expression of type II collagen in cartilage, which was reduced by OA induction. Conversely, MMP13 expression was significantly (*p* < 0.05) lower in the LMCP 200 group than in the ACLT group. These findings suggest that the protective effect of LMCP was related to the regulation of ECM synthesis and degradation in cartilage.

### Effect of LMCP on the mRNA Expression of Genes Related to ECM Synthesis in Chondrocytes Isolated from a Patient with OA

**Fig. 5:**
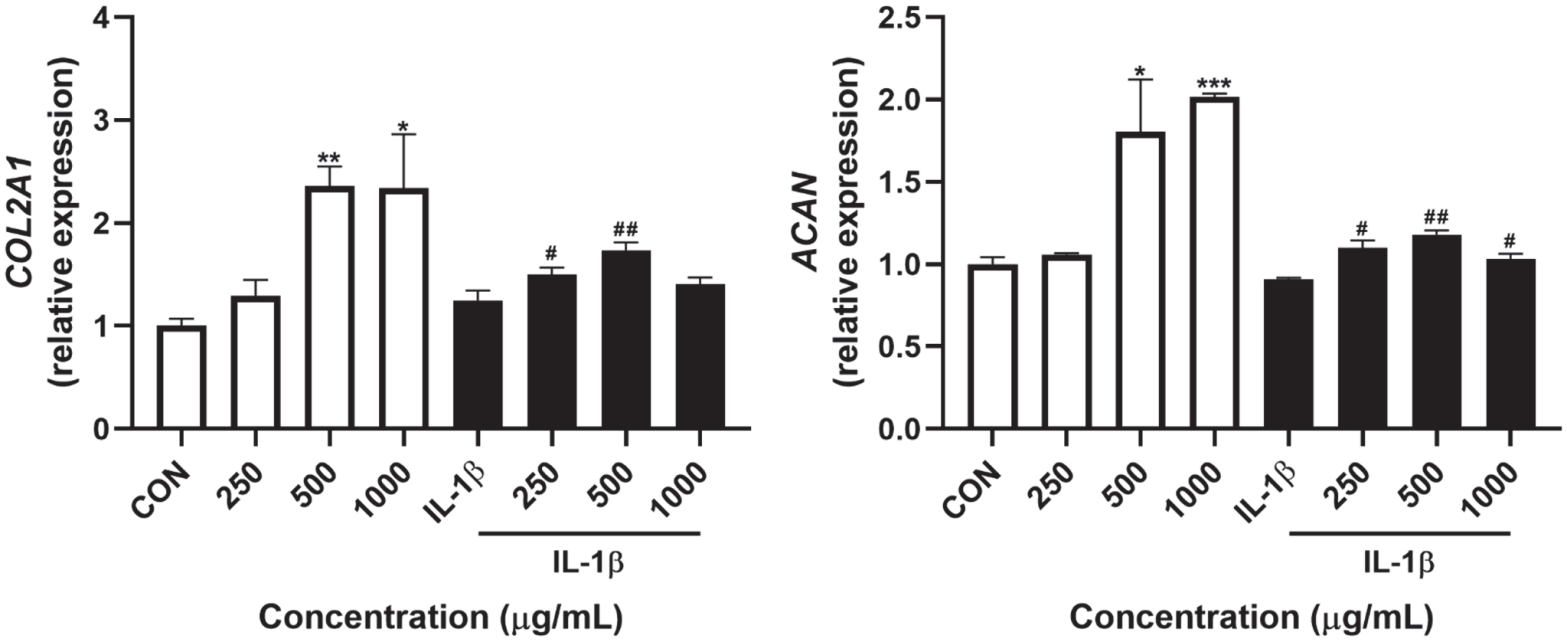
Effects of low-molecular-weight collagen peptide (LMCP) on the mRNA expression of genes related to extracellular matrix synthesis in chondrocytes isolated from a patient with OA. Data are presented as the mean ± SD (*n* = 3), and analysis was performed using a *t*-test. *p < 0.05, **p < 0.01, and ***p < 0.001 compared with the CON group. ^#^*p* < 0.05, and ^##^*p* < 0.01 compared with the IL-1β group.

According to the CCK-8 assay, LMCP was not cytotoxic to chondrocyte at concentrations up to 1,000 μg/ml (data not shown). Therefore, in vitro experiments were conducted using this concentration range. To confirm that LMCP promotes ECM synthesis in chondrocytes, the effects of LMCP on the mRNA expression of genes related to ECM were evaluated via qRT-PCR (Fig. 5). LMCP significantly increased the expression of *COL2A1* and *ACAN* in chondrocytes regardless of IL-1β induction. These results suggest that LMCP can effectively promote ECM synthesis during OA induction.

## Discussion

As the global population ages, the number of patients with OA continues to increase, making it ever more important to effectively treat or prevent OA. Because the analgesics and anti-inflammatory drugs used to treat OA can cause side effects, research to identify safe therapeutic ingredients such as animal and botanical extracts and methods to replace or reduce the intake of currently used treatments is underway [2-4]. In this study, we demonstrated that the oral administration of LMCP, a form of CH containing 3% Gly-Pro-Hyp with a tripeptide content exceeding 15%, had a protective effect on OA progression in the rabbit ACLT model of induced OA. In addition, our results indicated that the protective effect of LMCP originates from the promotion of chondrocyte ECM production by LMCP. The ECM of cartilage is mainly composed of type II collagen, which consists of homotrimers of the COL2A1 chain and large networks of proteoglycan containing glycosaminoglycan, hyaluronic acid, and chondroitin sulfate [25]. Cartilage ECM serves as a scaffold of cells and regulates the basic functions of cells including differentiation and survival [26]. Additionally, aggrecan, which is a major proteoglycan in cartilage, provides a hydrated gel structure that imparts the load-bearing properties of cartilage [27]. During OA progression, chondrocytes that synthesize ECM substances undergo many phenotypic changes in addition to apoptosis, resulting in an imbalance of ECM synthesis and degradation [28]. Because cartilage degradation accelerates OA, maintaining cartilage ECM and increasing ECM synthesis by chondrocytes are important for OA control. Our in vitro results illustrated that LMCP stimulated ECM synthesis by upregulating the mRNA expression of *ACAN* and *COL2A1* in chondrocytes from a patient with OA. Consistent with the in vitro results, LMCP increased the protein expression level of type II collagen and prevented the loss of proteoglycan in cartilage of the rabbit ACLT model. In line with our results, Naraoka *et al*. [11] reported that periodic knee injections of high-concentration tripeptide derived from CH ameliorated OA by increasing the synthesis of type II collagen in the cartilage of ACLT rabbits. Therefore, we predict that LMCP ameliorated OA progression by improved type II collagen and proteoglycan maintenance in cartilage by promoting the synthesis of type II collagen and aggrecan in chondrocytes.

OA progression is affected by both cartilage and subchondral bone damage. Although most researchers have focused on changes in cartilage in the early stage, some researchers reported that microstructural changes in subchondral bone may act as a primary pathologic process in OA [29]. In addition, bone damage both alters the underlying biomechanics of cartilage and negatively affects the interaction between chondrocytes and bone, which can accelerate OA progression [30]. Therefore, observing the subchondral bone damage that occurs during OA is important for assessing the severity of OA. In our micro-CT analysis, oral LMCP improved BV/TV, BS/BV, and TbTh in deteriorated tibial subchondral bone in the rabbit ACLT model, suggesting that LMCP can even help to retain the microstructure of subchondral bone, which is degraded during OA, by protecting cartilage. The improvement of the subchondral bone structure induced by LMCP could help control OA by ameliorating one of its primary pathologic processes. The bone-improving effect of CH has already been proven through the bone fraction-healing effect in a fracture rat model and the increase in bone density in postmenopausal women [10, 31]. Therefore, in this study, the possibility that LMCP protected cartilage by preferentially improving the structure of bone should not be excluded. Additionally, to our knowledge, this is the first study to confirm the positive effect of CH on OA-induced bone damage, and as such, these data are meaningful as new evidence that CH can ameliorate OA.

uring the pathogenesis of OA, injury to cartilage and bone tissue increases the levels of pro-inflammatory cytokines in synovial fluid such as IL-1β and IL-6, which play a critical role in OA development [32, 33]. Increased IL-1β and IL-6 levels upregulate the expression of ECM-degrading enzymes such as MMPs, thereby exacerbating OA [34]. Therefore, it is important to reduce the concentrations of pro-inflammatory cytokines in synovial fluid during OA progression. In our study, LMCP reduced the concentrations of IL-1β and IL-6 in synovial fluid and MMP13 expression in cartilage tissues in the rabbit ACLT model. We predict that the decreases in the concentrations of pro-inflammatory cytokines are related to the protective effects of LMCP on cartilage and subchondral bone. In addition, we presume that decreases in cytokine concentrations result in MMP13 downregulation. These results provide further evidence that LMCP has a therapeutic effect on OA progression.

In our previous study, LMCP exerted beneficial effects on skin health by stimulating skin fibroblasts and promoting the synthesis of ECM components such as type I collagen and hyaluronic acid [17, 18]. Similar to our previous findings, LMCP contributed to OA inhibition by promoting the synthesis of type II collagen and aggrecan in chondrocytes. This effect may very well be attributable to functional peptides such as Gly-Pro-Hyp and Pro-Hyp absorbed in the body following the oral administration of LMCP. Because Gly-Pro-Hyp and Pro-Hyp are the main peptides constituting type I and II collagen, cells present in collagen-rich tissues could be activated by Gly-Pro-Hyp and Pro-Hyp. In addition, Gly-Pro-Hyp and Pro-Hyp can act as inducers to increase ECM levels in cells. Previous reports revealed that Gly-Pro-Hyp and Pro-Hyp increased the growth of osteoblasts and skin fibroblasts while also exerting chemotactic effects on fibroblasts such as stimulating their migration [12, 13, 35]. Collectively, functional peptides including Gly-Pro-Hyp and Pro-Hyp that were absorbed into the blood following oral LMCP administration may have activated chondrocytes and inhibited OA in the rabbit ACLT model. However, this is a prediction and cannot be accurately determined in the current study. In addition, the protective effect on OA progression by peptides other than Gly-Pro-Hyp and Pro-Hyp cannot be excluded. Therefore, further studies are required to more accurately reveal which peptides in LMCP mainly act to ameliorate OA progression. Studying the protective effect on OA progression and pharmacokinetics using a single substance of the major peptides present in LMCP will help to gain deeper insight into the protective effect of LMCP on OA progression.

In conclusion, oral LMCP prevented OA progression in the rabbit ACLT model. LMCP supplementation ameliorated cartilage and subchondral bone damage by promoting type II collagen and aggrecan synthesis in chondrocytes. Additionally, the reductions of the concentrations of pro-inflammatory cytokines in synovial fluid and MMP13 expression in cartilage further demonstrated that LMCP effectively inhibited OA. These findings indicate that LMCP has potential as a safe therapeutic ingredient for OA that stimulates cartilage regeneration.

## Figures and Tables

**Table 1:** Primer sequences.

Gene	Forward (5′→3′)	Reverse (5′→3′)
*COL2A1*	TCT ACCCCAATCCAGCAAAC	GTTGGGAGCCAGATTGTCAT
*ACAN*	TGTGGGACTGAAGTTCTTGG	AGCGAGTTGTCATGGTCTG
